# Monocyte-dependent co-stimulation of cytokine induction in human γδ T cells by TLR8 RNA ligands

**DOI:** 10.1038/s41598-021-94428-6

**Published:** 2021-07-27

**Authors:** Ruben Serrano, Christoph Coch, Christian Peters, Gunther Hartmann, Daniela Wesch, Dieter Kabelitz

**Affiliations:** 1 Institute of Immunology, Christian-Albrechts University of Kiel and University Hospital Schleswig-Holstein Campus Kiel, Arnold-Heller-Str. 3, Building U30, 24105 Kiel, Germany; 2 Department of Clinical Chemistry and Clinical Pharmacology, University of Bonn, Bonn, Germany

**Keywords:** Immunology, Immunotherapy, Innate immunity, Lymphocytes, Translational immunology

## Abstract

Human Vγ9Vδ2 T cells recognize pyrophosphates produced by microbes and transformed cells and play a role in anti-infective immunity and tumor surveillance. Toll-like receptors (TLR) are pattern recognition receptors in innate immune cells which sense microbial structures including nucleic acids. Given that γδ T cells are in clinical development for application in cellular cancer immunotherapy and TLR ligands have potent adjuvant activity, we investigated the co-stimulatory role of selected TLR ligands in γδ T-cell activation. Here we have used recently described RNA ligands for TLR7 and TLR8 together with Vγ9Vδ2 T-cell specific pyrophosphate antigens to analyze the rapid cytokine induction in Vδ2 T cells as well as the accessory cell requirements. While TLR8- as well as TLR7/8-specific RNA did not induce IFN-γ in Vδ2 T cells on their own, they provided strong co-stimulation for Vδ2 T cells within peripheral blood mononuclear cells in the presence of additional T-cell receptor activation. In contrast, TLR7 ligands were ineffective. Purified γδ T cells did not directly respond to TLR8 co-stimulation but required the presence of monocytes. Further experiments revealed a critical role of IL-1β and IL-18, and to a slightly lesser extent of IL-12p70, in the co-stimulation of Vδ2 T cells by TLR8 and TLR7/8 RNA ligands. Results of intracellular cytokine expression were validated by ELISA analysis of cytokines in cell culture supernatants. The cell context-dependent adjuvant activity of TLR8 and TLR7/8 RNA ligands described here might be important for the future optimization of γδ T-cell based cancer immunotherapy.

## Introduction

Approximately 2–5% of CD3^+^ T cells in the peripheral blood carry a γδ T-cell receptor (TCR) rather than the conventional αβ TCR. In the blood of adult donors, the majority of γδ T cells express Vγ9 paired with Vδ2, for simplicity referred to as Vδ2 throughout this study^1,2^. Vδ2 T cells recognize phosphorylated metabolites of isoprenoid biosynthesis required for cholesterol synthesis and protein prenylation. Such pyrophosphates, collectively termed phosphoantigens (pAg), are intermediates of the non-mevalonate pathway used by many bacteria and parasites, but are also generated in the eukaryotic mevalonate pathway. Microbial pAg such as (*E*)-4-Hydroxy-3-methyl-but-2-enyl pyrophosphate (HMBPP) stimulate human Vδ2 T cells at nanomolar concentrations, whereas micromolar concentrations of eukaryotic pAg like isopentenyl pyrophosphate (IPP) are required for activation of the very same Vδ2 T cells^3,4^. Activation of Vδ2 T cells by microbial or eukaryotic pAg requires the presence of butyrophilin (BTN) transmembrane molecules, specifically BTN3A1 and BTN2A1^5–7^. Healthy cells produce insufficient amounts of the pAg IPP to activate γδ T cells. Many transformed cells, however, have a dysregulated mevalonate pathway leading to increased accumulation of IPP, which then triggers the activation of Vδ2 T cells^8^. As a consequence, many solid tumors and leukemias/lymphomas are recognized and killed by γδ T cells. Importantly, the sensitivity of tumor cells to lysis by Vδ2 T cells can be strongly increased by nitrogen-containing aminobisphosphonates like zoledronate which interfere with the mevalonate pathway leading to accumulation of IPP^9^. Zoledronate is also a potent and selective stimulus for Vδ2 T-cell expansion when added to peripheral blood mononuclear cells (PBMC), due to the zoledronate-triggered production of IPP in monocytes^10^. Given the additional advantage of their HLA-independence (i.e., application across HLA barriers is possible^11^), there is an increasing interest to apply γδ T cells in cancer immunotherapy^12–14^.

The activation and differentiation of γδ T cells is regulated by co-stimulatory signals and the cytokine milieu. While human γδ T cells have the capacity to acquire functional phenotypes resembling Th1, Th2, Th9, Th17 cells and others, ex vivo isolated Vδ2 T cells are usually prone to produce mainly interferon-γ (IFN-γ) and tumor necrosis factor-α (TNF-α)^15–17^. The effector activity of γδ T cells is modulated by innate immune cells like monocytes, macrophages and dendritic cells (DC) activated by multiple pattern recognition receptors. In this regard, Toll-like receptors (TLR) play a central role as sensors of microbial products, resulting in the context-dependent production of pro-inflammatory and anti-inflammatory cytokines, or type I interferons. Not surprisingly, γδ T-cell activation and differentiation is also modulated by TLR ligands^18,19^. γδ T cells themselves can express certain TLR, and direct effects of some TLR ligands on human and mouse γδ T-cell activation have been reported^20–23^.

We recently studied possible effects of TLR7 and TLR8 ligands on the activation of human Vδ2 T cells. Both TLR7 and TLR8 are endosomal receptors and recognize single-stranded microbial RNA as their natural ligand^24^. However, for both receptors non-RNA ligands mainly of the imidazoquinoline compound class have been described, such as Imiquimod (TLR7) and Resiquimod (TLR7/8)^25^. We observed that Resiquimod (but not Imiquimod) per se stimulated some IFN-γ production in Vδ2 T cells within the total population of PBMC, and co-stimulated IFN-γ production in response to the Vδ2 T-cell specific pAg HMBPP. At the same time, non-RNA TLR8 ligands like Resiquimod or Motolimod inhibited the proliferative expansion of Vδ2 T cells in the presence of monocytes^26^. Mechanistically, we found that a substantial proportion of monocytes died in response to the imidazoquinoline TLR ligands, thereby providing strong co-stimulation for γδ T-cell IFN-γ production but insufficient support for their cellular expansion^26^.

γδ T cells are considered to share features of innate and adaptive immunity. Due to their independence of antigen processing for TCR-mediated responsiveness, their activation is rapidly modulated by signals from innate immune cells. To investigate the modulation of γδ T-cell activation by TLR7 and TLR8 ligands more physiological than imidazoquinoline compounds, we made use of RNA ligands for TLR7 and TLR8 mimicking viral ssRNA^27–29^ to re-evaluate the role of these TLR in Vδ2 T-cell activation, with a focus on the role of monocytes and selected cytokines.

## Materials and methods

### Isolation of cell populations

Leukocyte concentrates of healthy adult blood donors were obtained from the Institute of Transfusion Medicine, University Hospital Schleswig–Holstein (Kiel, Germany). Written informed consent was obtained from all donors. The research was conducted in accordance with the Declaration of Helsinki and was approved by the relevant institutional review boards (ethic committee of the Medical Faculty of the University of Kiel, Germany; code number: D546/16). PBMC were isolated by Ficoll-Hypaque density gradient centrifugation. γδ T cells were purified from PBMC by positive magnetic selection using the anti-TCRγ/δ micro-Bead Kit from Miltenyi Biotec (Bergisch Gladbach, Germany) following the instructions of the company. To increase the purity of γδ T cells, two consecutive magnetic columns were applied. The purity of isolated γδ T cells was > 97%. Monocytes were isolated from PBMC by negative isolation using the Pan Monocyte Isolation Kit (Miltenyi Biotec) or the EasySep monocyte isolation kit (Stem Cell Technologies, Cologne, Germany) following the instructions of the companies. Negatively isolated monocytes contained > 92% CD14^+^ monocytes and < 0.2% contaminating CD3^+^ T cells as determined by flow cytometry.

### Reagents

The following reagents were obtained from Invivogen (Toulouse, France): TL8-506 (TLR8 ligand), Resiquimod (TLR7/8 ligand), ssRNA40 (TLR8 RNA ligand), ssRNA41 (inactive control for ssRNA40), CU-CPT9a (TLR8 inhibitor)^30^, neutralizing anti-IL-1β antibody (mabg-hil1b-3), neutralizing anti-IL-18 antibody (mabg-hil18-3). Neutralizing anti-IL-12p70 antibody (MAB219) was purchased from R&D Systems (Bio-Techne, Wiesbaden, Germany). Human recombinant cytokines were obtained as follows: IL-1β and IL-12 were from ImmunoTools (Friesoythe, Germany), IL-18 from MBL (Biozol, Eching, Germany), IL-2 (Proleukin) was kindly provided by Novartis (Basel Switzerland). The TLR8 agonist Motolimod (VTX-2337) was purchased from Sellekchem (Houston, TX, USA). Phytohemagglutinin (PHA) was obtained from Sigma Aldrich (Schnelldorf, Germany). The Vγ9Vδ2-selective pAg (*E*)-4-Hydroxy-3-methyl-but-2-enyl pyrophosphate (HMBPP) was purchased from Echelon Biosciences (Salt Lake City, UT, USA). The following RNA ligands for TLR 7 and TLR8 have been previously described^27–29^ and were synthesized by Biomers.net (Ulm, Germany): R805 (TLR7), R2152 and R2196 (TLR8), 9.2s (TLR7/8), polyCA (negative inert control RNA). RNA ligands were complexed with low MW poly-arginine (pArg; Sigma Aldrich) before adding to cell cultures. In control experiments, the maximal final concentration of pArg (2 µg/mL) did not exert any effect on γδ T-cell activation. Transfectants expressing TLR7 (HEK-blue-hTLR7; hkb-htlr7), TLR8 (HEK-blue-hTLR8; hkb-htlr8) were obtained from Invivogen.

### Cell cultures

PBMC (4 × 10^5^ per well) or purified γδ T cells (0.5–1 × 10^5^ per well) with or without purified monocytes (1 × 10^5^ per well) were cultured in wells of 96-well round bottom microtiter plates (Nunc; Thermo Fisher Scientific, Waldham, MA, USA). Complete culture medium was RPMI 1640 (Thermo Fisher Scientific) supplemented with antibiotics (100 U/mL penicillin, 100 µg/mL streptomycin) and 10% of heat-inactivated low endotoxin fetal bovine serum (Bio&Sell, Feucht, Germany). Where indicated, cell cultures were supplemented with 10 ng/mL of IL-1β, 10 ng/mL of IL-12, 20 ng/mL of IL-18, or 5 µg/mL of neutralizing anti-cytokine antibodies. Vγ9Vδ2 T-cell lines were established from PBMC containing 2–3% CD3^+^Vδ2^+^ γδ T cells as described^31^. Briefly, PBMC were stimulated with 2.5 μM zoledronate (Novartis) and 50 IU/mL IL-2. IL-2 was added every other day, and cultures were split on day 7 and day 9. After 12 days, such γδ T-cell lines contained > 90% CD3^+^Vδ2^+^ γδ T cells. γδ T-cell lines were seeded at 2 × 10^5^ cells per well in complete medium supplemented with 10 IU/mL IL-2. Cell cultures were stimulated or not with TLR ligands in the absence or additional presence of 1 nM HMBPP, and were incubated for 24 h at 37^0^C in a humidified atmosphere of 5% CO_2_ in air. For measurement of intracellular cytokines, 3 μM monensin was added during the last 4 h to prevent cytokine secretion.

### Flow cytometry

The following mAb were obtained from BD Biosciences (Heidelberg, Germany): anti-CD3-PE/APC/BV605 (clone SK7), anti-CD14-FITC/APC (clone MoP9), anti-IFN-γ-PE or -PE/Cy7 (clone 4S.B3), anti-Granzyme B-PE-CF954 (clone GB11), anti-TNF-α-PE (clone MAb 11). Anti-Vδ2-FITC (clone IMMU389) was obtained from Beckman Coulter (Krefeld, Germany), anti-hTLR7-PE and anti-hTLR8-AF647 were purchased from R&D Systems. For cell surface staining, cells were washed, stained for 20 min on ice with mAb, washed twice, and resuspended in 1% paraformaldehyde. For intracellular staining, cells were permeabilized in Cytofix/Cytoperm buffer (BD Biosciences) before staining with fluorochrome-conjugated mAb. Final antibody concentrations were used according to respective data sheets. All analyses were measured on a FACS Calibur or LSR Fortessa cytometer (BD Biosciences), using the software CellQuest Pro and DIVA (Data-Interpolating Variational Analysis) for acquisition respectively, and FlowJo v10.6.1 software for data analysis.

### Quantification of cytokines in cell culture supernatants

IFN-γ and Granzyme-B (GrB) in cell culture supernatants were quantified using Human IFN-γ (DY285B) or GrB (DY2906) DuoSet ELISA kits from R&D Systems, following the instructions of the company. Cytokines including TNF-α, IL-1β, IL-12p70 and IL-18 were measured by Luminex Multiplex assay (R&D Systems) according to the instructions of the company.

### Statistical analysis

Statistical comparisons were made between groups using one-or two-way ANOVA analysis and Dunnett´s multiple comparison test against respective internal controls. All analyses were done with the Graphpad Prism 8 software. Levels of significance were set as * p < 0.05, ** p < 0.01, *** p < 0.001, and **** p < 0.0001.

## Results

### Expression of TLR7 and TLR8

**Figure 1:**
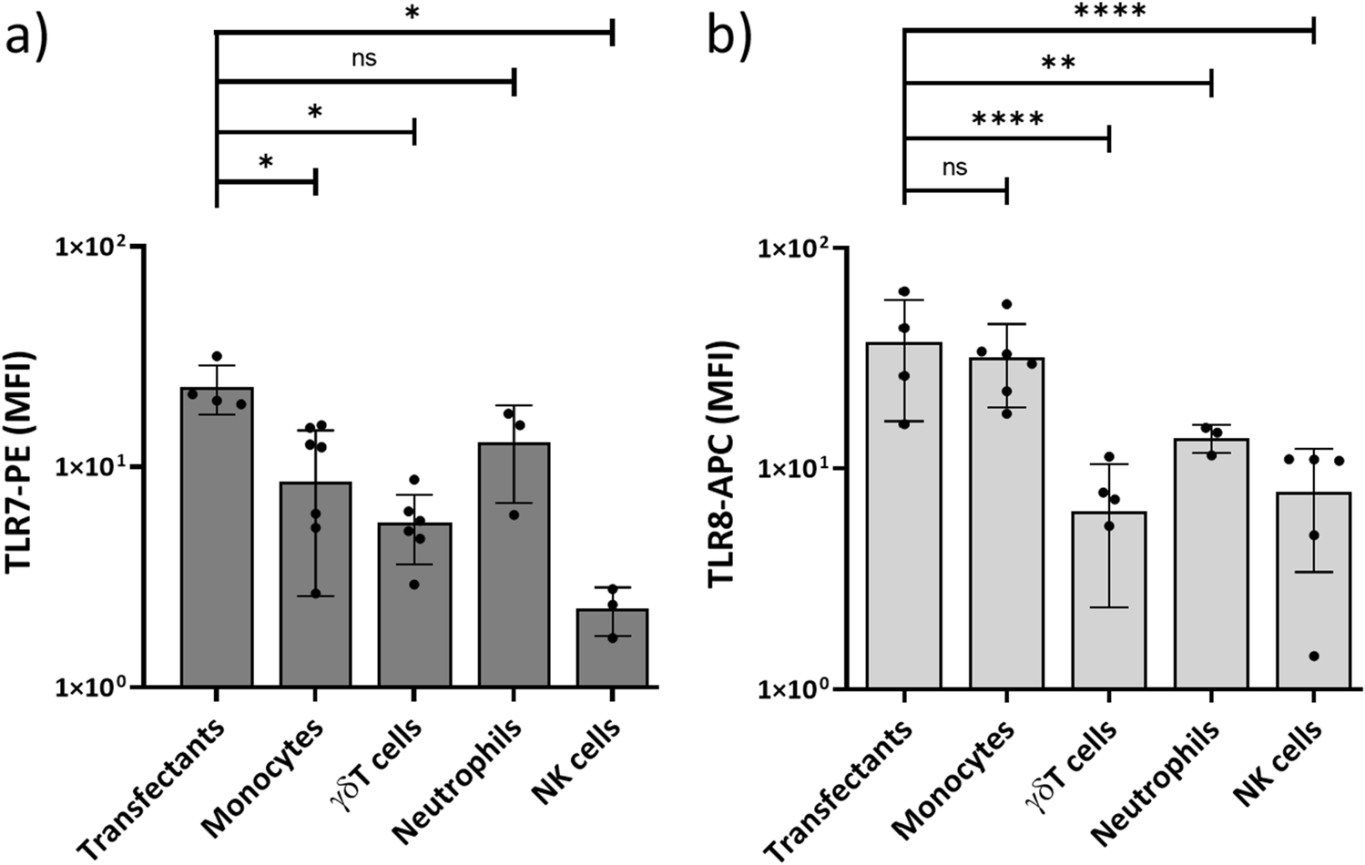
Expression of TLR7 and TLR8 in immune cells. Purified monocytes, γδ T cells, neutrophils and NK cells, as well as TLR7- and TLR8-transfected HEK cells were stained for intracellular analysis of TLR7 and TLR8 expression. Results are depicted as mean ± SD of median fluorescence intensity (MFI) of specific antibody staining of three to seven experiments with different healthy blood donors. (**a**) TLR7, (**b**) TLR8. Statistical significance is indicated with * p < 0.05, ** p < 0.01, **** p < 0.0001. ns, not significant.

We analyzed expression patterns of TLR7 and TLR8 by flow cytometry in peripheral blood leukocyte subsets, i.e. monocytes, γδ T cells, neutrophils as well as NK cells. For comparison, we used HEK cells stably transfected with TLR7 or TLR8, respectively. As shown in a representative experiment (Suppl. Fig. S1), TLR7 was weakly expressed, with monocytes and neutrophils expressing slightly higher levels than γδ T cells and NK cells. TLR8 was detected in monocytes, neutrophils and NK cells, in line with published studies on the functional expression of TLR8 in these cells^32–36^. In this donor, γδ T cells expressed very low levels of TLR8 as determined by Δ MFI (MFI TLR8 minus MFI isotype control). A summary of several experiments performed with different healthy blood donors is presented for TLR7 in Fig. 1a and for TLR8 in Fig. 1b. Here, MFI of isotype controls have not been subtracted. Overall, monocytes expressed same levels of TLR8 like the HEK-TLR8 transfectants, and both expressed significantly higher levels of TLR8 in comparison to other analyzed immune cell populations.

### Co-stimulatory activity of TLR8 and TLR7/8 but not TLR7 RNA ligands

PBMC were activated with various TLR subset-specific RNA ligands and for comparison with well characterized non-RNA small molecule ligands, in the absence or presence of the Vγ9Vδ2 T-cell specific phosphoantigen HMBPP for simultaneous TCR activation. After 24 h, intracellular expression of IFN-γ was analyzed by flow cytometry with a gate set on Vδ2 T cells. Results presented in Suppl. Fig. S2 indicate that neither TLR7-specifc RNA pG805 (as well as the inactive control RNA pCA^37^) nor TLR7-specific imidazoquinoline ligand Imiquimod had any co-stimulatory activity (TLR ligands without HMBPP: gray histograms; TLR ligands together with HMBPP: open histograms). In contrast, non-RNA TLR8 ligands TL8-506 and Motolimod significantly enhanced the weak HMBPP-induced IFN-γ expression (Suppl. Fig. S3). Two different TLR8-specific RNA ligands (ssRNA40, R2152) strongly co-stimulated the HMBPP-induced IFN-γ expression, as did the TLR7/8-specific RNA 9.2s, while a third TLR8-specific RNA (R2196) was less active (Suppl. Fig. S3). The well-established imidazoquinoline TLR7/8 agonist Resiquimod was less active when compared to 9.2s, mainly due to different kinetics (see below). Overall, TLR8 RNA ligands strongly enhanced IFN-γ in Vδ2 T cells in the presence of the TCR stimulus HMBPP but showed only weak activity and only at higher concentrations (2–4 µg/mL) in the absence of HMBPP (compare gray histograms in Suppl. Fig. S3). Based on the dose titrations presented, we selected optimal concentrations for subsequent experiments (4 μg/mL R2152 and 9.2s, 2 μg/mL ssRNA40 and Resiquimod).

**Figure 2:**
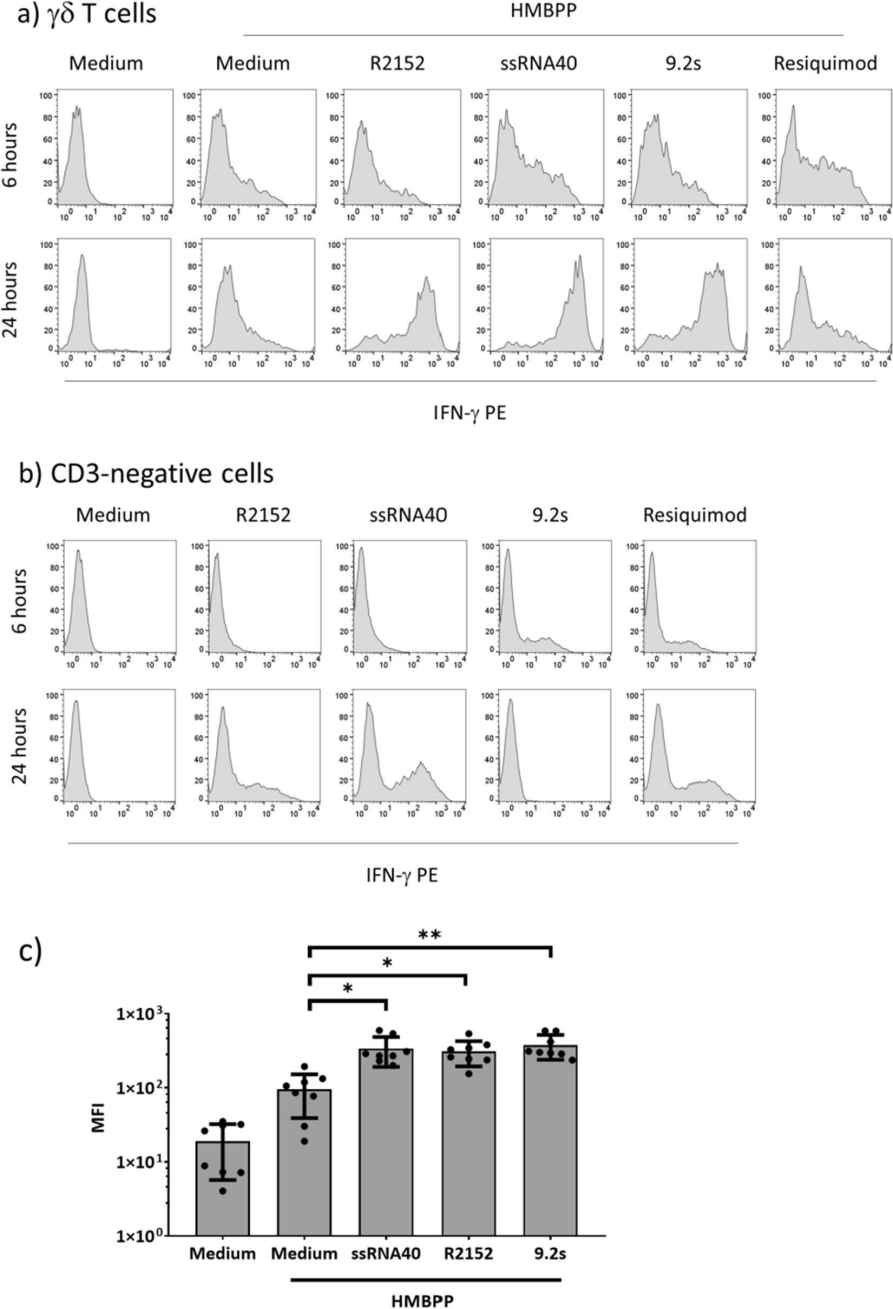
Kinetics of IFN-γ induction in γδ T cells and CD3-negative lymphocytes. PBMC were cultured in the presence or absence of 1 nM HMBPP and the indicated TLR ligands. After 6 and 24 h, the intracellular expression of IFN-γ was analyzed with a gate set on (**a**) CD3^+^Vδ2^+^ γδ T cells, or (**b**) CD3-negative lymphocytes (i.e., mainly NK cells). Representative histograms are shown in (**a**) and (**b**). A summary of eight experiments is presented in (**c**). Results are expressed as mean fluorescence intensity (MFI) ± SD. Statistical significance is indicated with * p < 0.05, ** p < 0.01.

**Figure 3:**
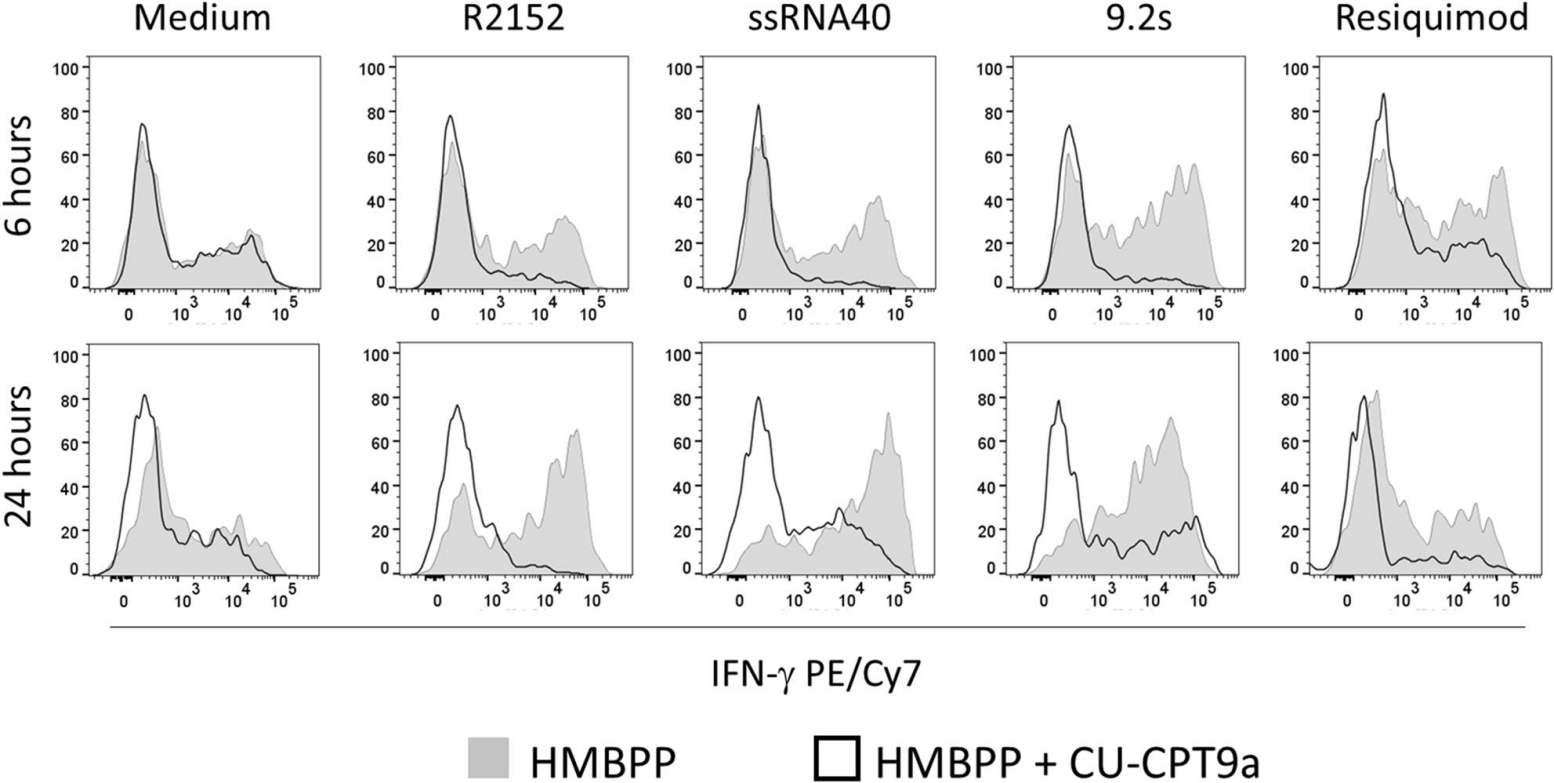
Co-stimulation by RNA ligands is TLR8-dependent. PBMC were activated with HMBPP in the presence or absence of TLR8 ligands and additional presence or absence of 10 µM TLR8 inhibitor CU-CPT9a. Intracellular expression of IFN-γ in CD3^+^Vδ2^+^ γδ T cells was analyzed after 6 (upper panel) and 24 h (lower panel). Gray histograms: without CU-CPT9a, open histograms: with CU-CPT9a. One out of two experiments with similar results is shown.

Next, we performed time course studies to follow the co-stimulatory effect of TLR8 ligands over time. In these experiments, PBMC were cultured for 6 and 24 h in the presence or absence of TLR ligands and HMBPP, and intracellular IFN-γ expression was measured within Vδ2 γδ T cells (Fig. 2a) and within CD3-negative cells in the lymphocyte gate (Fig. 2b). Figure 2a illustrates that Resiquimod exerted higher co-stimulation on HMBPP-activated γδ T cells at the earlier time point when compared to 24 h, whereas all three TLR8 and TLR7/8 RNA ligands were much more active at 24 h. In line with published reports^34,38^, we also observed IFN-γ expression induced by TLR ligands only (i.e., in the absence of HMBPP) within CD3-negative lymphocytes, most likely NK cells (Fig. 2b). The potent co-stimulatory activity of TLR8 RNA ligands on the IFN-γ induction in Vδ2 T cells within PBMC activated for 24 h with HMBPP was validated in a total of eight donors (Fig. 2c). Moreover, the co-stimulatory effect of the TLR ligands was clearly dependent on TLR8, as shown by the effect of the selective inhibitor CU-CPT9a^30^ at both 6 and 24 h of activation (Fig. 3).

### Role of endogenous cytokines

**Figure 4:**
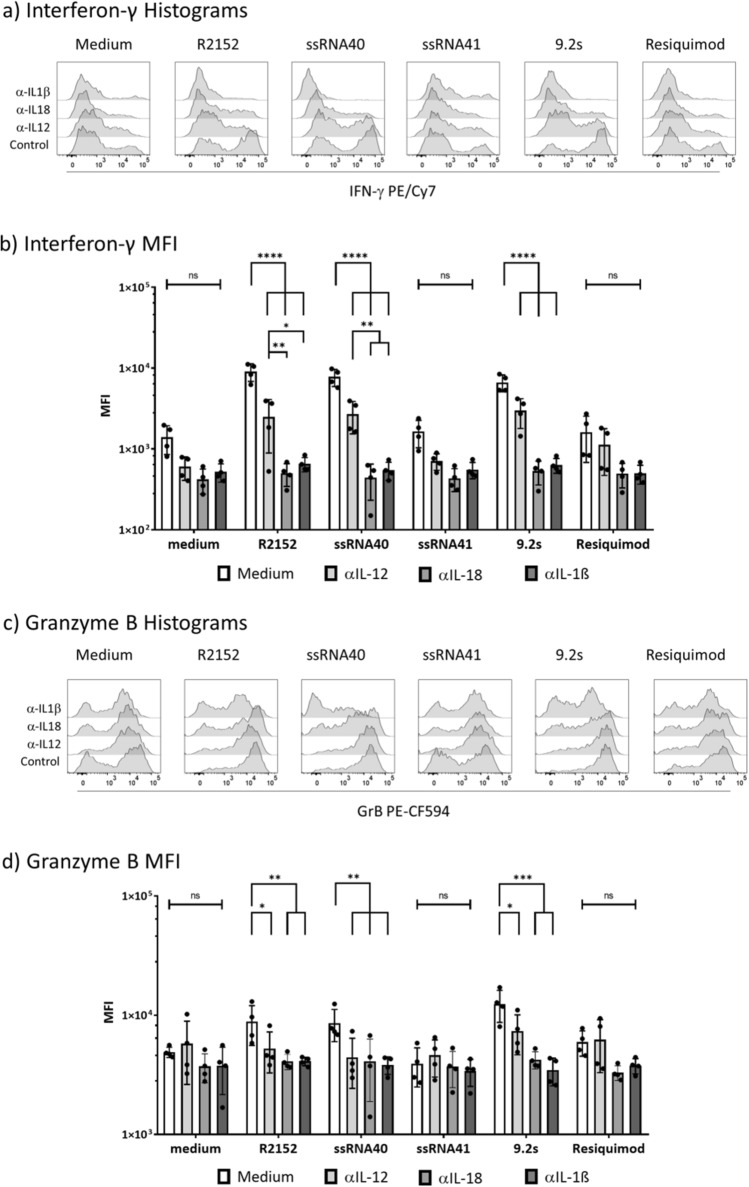
Modulation of cytokine induction in Vδ2 T cells by anti-cytokine antibodies. PBMC were activated with HMBPP in the presence or absence of TLR8 ligands and additional presence or absence of 5 µg/mL of neutralizing anti-cytokine antibodies as indicated. Intracellular expression of IFN-γ (**a**,**b**) and GrB (c,d) in CD3^+^Vδ2^+^ γδ T cells was analyzed after 24 h. The lower histograms in each panel of (**a**) and (**c**) show the upregulated cytokine expression in the absence of anti-cytokine antibodies (“controls”). (**a**), (**c**) representative histograms; (**b**), (**d**) Summary of four experiments. Results are expressed as mean fluorescence intensity on a logarithmic scale (MFI) ± SD. Statistical significance is indicated with * p < 0.05, ** p < 0.01, *** p < 0.001, **** p < 0.0001. ns, not significant.

In the experiments presented so far, we described the co-stimulatory effect of TLR8 RNA ligands on the IFN-γ induction in Vδ2 T cells within PBMC stimulated with the pAg HMBPP. Monocytes comprise a substantial proportion of PBMC and are known to express TLR8 (Fig. 1) and to produce a range of cytokines in response to TLR8 activation, some of which are important regulators of T-cell differentiation^28,32–34,38,39^. In the context of γδ T-cell activation, IL-1β, IL-12p70 and IL-18 are of particular interest. IL-1β has multiple effects in early T-cell activation^40^, and IL-18 is a crucial factor in the regulation of IFN-γ induction^41^. IL-12p70 on the other hand is a key cytokine in the differentiation of Th1 T cells^32^ which is the primary differentiation pathway of ex vivo isolated human γδ T cells^42^. We have previously reported that monocytes secrete IL-1β in response to TLR8 ligands ssRNA40 and Resiquimod^26^. Together with IL-1β and TNF-α, we also detected IL-12p70 and IL-18 in the supernatants of purified monocytes activated by TLR8 RNA ligand ssRNA40 (as well as by Resiquimod) (Supplemental Table 1).To address the potential contribution of these cytokines, we added neutralizing antibodies against IL-1β, IL-18 and IL-12p70 to PBMC activated with HMBPP in the absence or presence of TLR8 ligands. Again, the intracellular cytokine expression in Vδ2 T cells was measured after 24 h. In addition to IFN-γ we also analyzed Granzyme B (GrB) which is a major effector molecule of cytotoxic γδ T cells^43^. Results of a representative experiment are depicted in Fig. 4a (IFN-γ) and Fig. 4c (GrB). The expression of the cytokines in the absence of added antibodies (control) is shown in the lowest histogram for both cytokines. As before, there was a clear costimulatory effect on IFN-γ and GrB induction by TLR8 ligands R2152 and ssRNA40, and by TLR7/8 ligand 9.2s, but not by the inactive control ssRNA41 (Fig. 4a,c). In contrast to IFN-γ (and TNF-α; not shown), a major proportion of Vδ2 T cells already expressed GrB in the absence of added TLR8 ligands, but the active ligands (and not the inactive control ssRNA41) clearly further co-stimulated GrB expression (Fig. 4c). The addition of neutralizing anti-cytokine antibodies reduced (anti-IL-12p70) or abolished (anti-IL-1β, anti-IL-18) the co-stimulatory effect of TLR8 RNA ligands on IFN-γ (Fig. 4a) and TNF-α (not shown) production. While the HMBPP-induced GrB expression in Vδ2 T cells (“Medium” in Fig. 4c) was not affected by anti-cytokine antibodies, the additional co-stimulatory effect of the TLR8 ligands was also abolished, most notably with anti-IL-1β. A summary of four experiments displaying the mean fluorescence intensity (MFI) and statistical analysis is shown for IFN-γ in Fig. 4b and for GrB in Fig. 4d. Note that MFI is displayed on a logarithmic scale, and the effect of all three anti-cytokine antibodies was highly significant (p < 0.0001) for IFN-γ. Taken together, these results indicate that all three cytokines contribute to the co-stimulation of cytokine induction in Vδ2 T cells by TLR8 RNA ligands within PBMC.

### Purified γδ T cells require monocytes for co-stimulation by TLR8 RNA ligands

**Figure 5:**
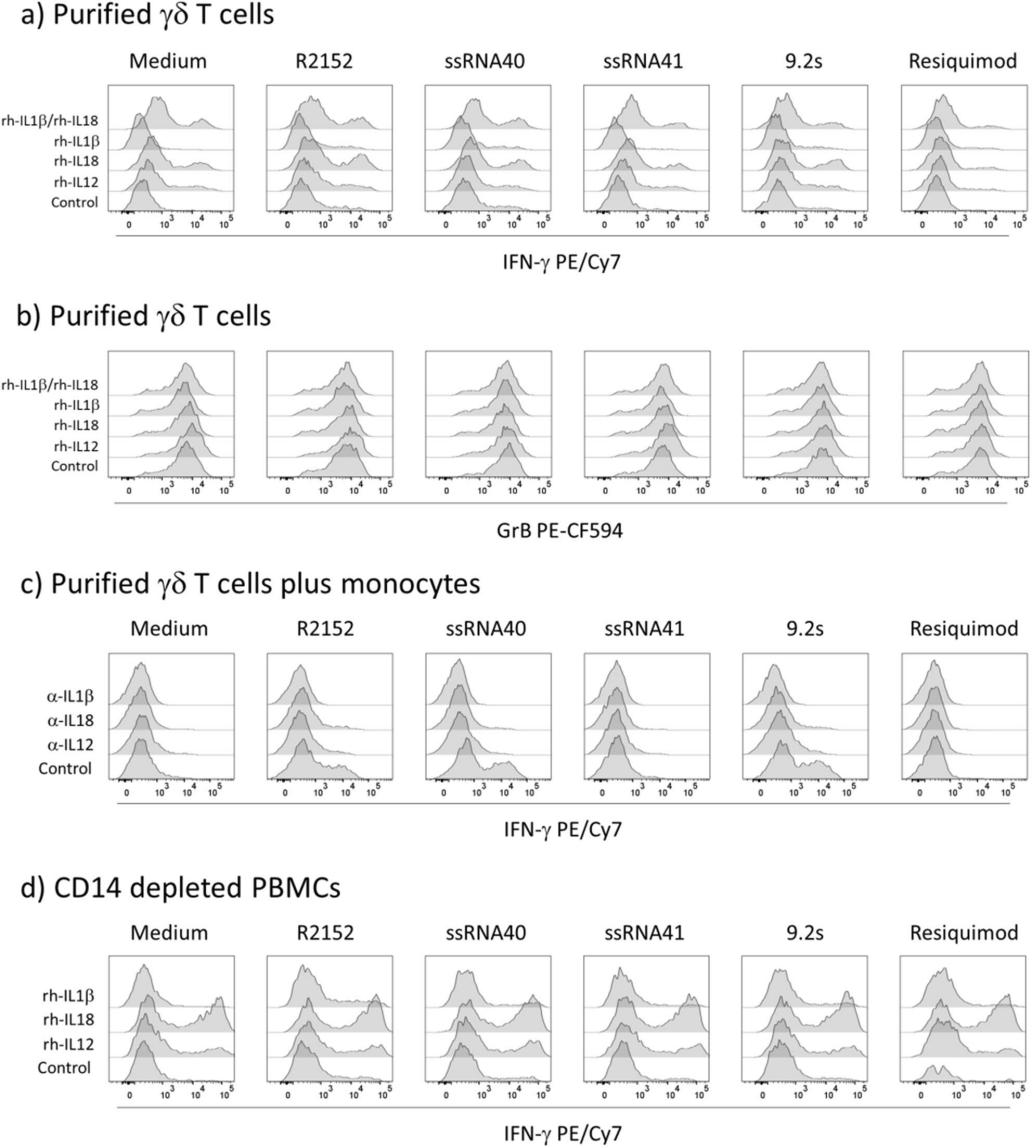
Role of monocytes in co-stimulation of γδ T cells by TLR8 ligands. 100.000 purified γδ T cells alone (**a**,**b**), or together with 100.000 purified monocytes (**c**), or 400.000 CD14-depleted PBMC (**d**) were activated with HMBPP in the presence or absence of TLR8 ligands as indicated. Recombinant cytokines IL-1β (10 ng/mL), IL-12 (10 ng/mL), IL-18 (20 ng/mL (**a**,**b**,**d**) or 5 µg/mL of neutralizing anti-cytokine antibodies (**c**) were added as indicated. Intracellular expression of IFN-γ (a,c,d) and GrB (**b**) in CD3^+^Vδ2^+^ γδ T cells was analyzed after 24 h. The lower histograms in each panel show the IFN-γ/GrB expression in the absence of added cytokines (**a**,**b**,**d**) or anti-cytokine antibodies (**c**). Histograms of one representative out of five (**a**,**b**,**c**) or two (**d**) experiments are shown.

**Figure 6:**
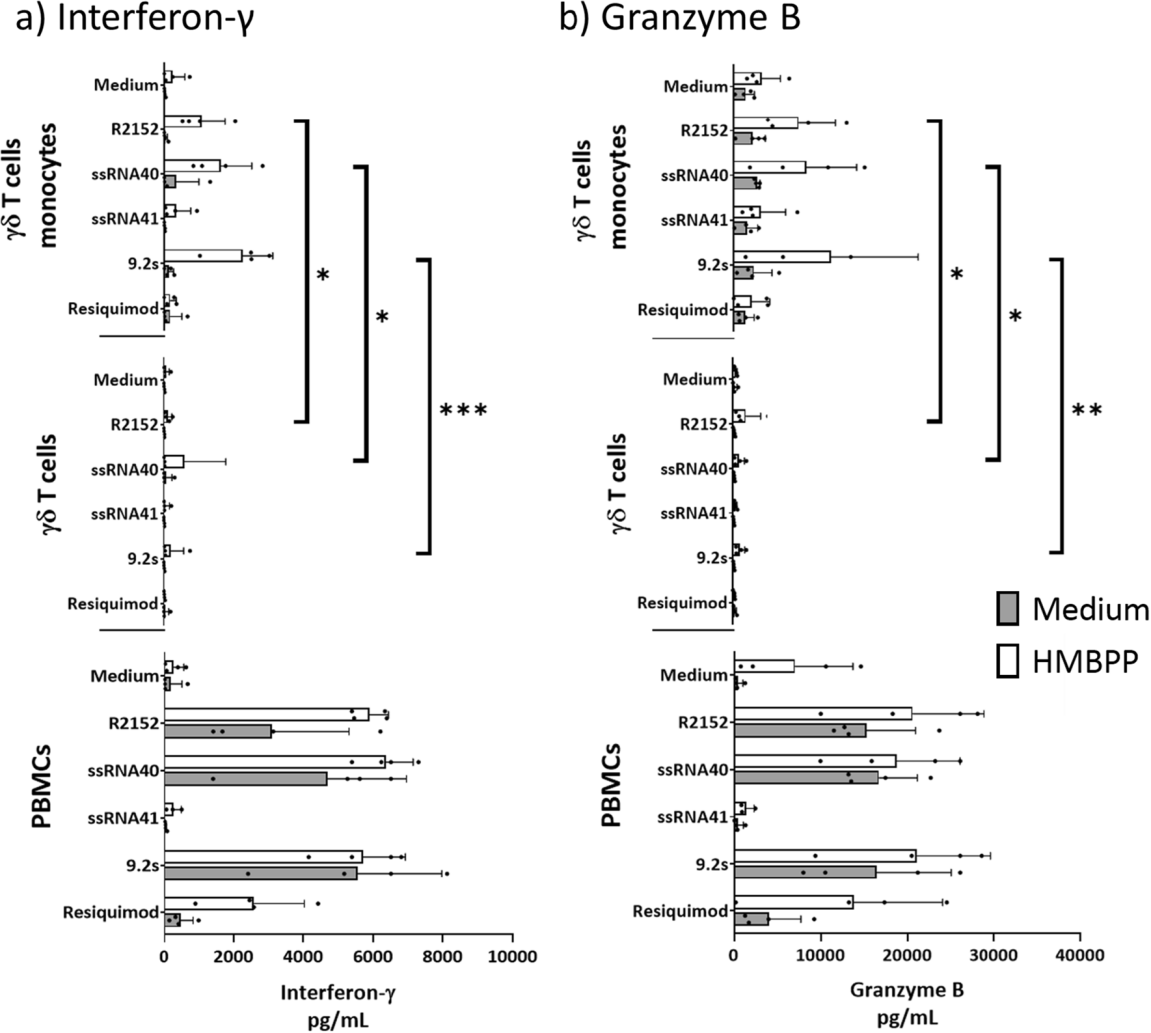
Co-stimulation of purified γδ T cells by TLR8 RNA ligands requires monocytes and TCR activation. 400.000 PBMC, 100.000 purified γδ T cells, and 100.000 purified γδ T cells reconstituted with 100.000 monocytes, were cultured in the absence (gray columns) or presence of HMBPP (open columns) and the absence or additional presence of TLR8 ligands. After 24 h, cell culture supernatants were collected and analyzed by ELISA for IFN-γ (**a**) and GrB (**b**). All ELISAs were performed on two independent replicates in each experiment. Mean ± SD of three to four independent experiments are shown. Statistical significance is indicated with * p < 0.05, ** p < 0.01, *** p < 0.001.

Next, we investigated the potential responsiveness of freshly isolated γδ T cells to co-stimulation with TLR8 RNAs. To this end, γδ T cells were purified by magnetic MACS sorting and stimulated for 24 h with HMBPP in the absence or presence of TLR8 ligands and recombinant cytokines IL-1β, IL-12p70, or IL-18. Under these conditions, no substantial induction of IFN-γ (Fig. 5a) or TNF-α (not shown) was observed, except for IL-18 which had a minor effect on IFN-γ induction, which was not further increased by the additional presence of IL-1β (Fig. 5a, top histograms). Again, high expression of GrB was also detected in the purified γδ T cells, which however was neither modulated by TLR8 ligands nor by the added cytokines (Fig. 5b). Together, these results indicate that purified γδ T cells are not directly co-stimulated by TLR8 RNA ligands, in line with the low TLR8 expression detected by flow cytometry. However, when monocytes isolated by negative MACS selection were added back, the co-stimulatory effect of R2152, ssRNA40 and 9.2s was restored as shown in the “control” histograms of a representative experiment in Fig. 5c. The co-stimulatory function of monocytes is at least partly due to TLR8-induced secretion of IL-1β, IL-18 or IL-12p70 as suggested by the blocking effect of the respective neutralizing antibodies (Fig. 5c). IL-18 is a key factor for IFN-γ induction^41^ (in fact it has been initially described as “γ-Interferon-inducing factor”). While the data in Fig. 5a indicated that IL-18 does not directly affect IFN-γ induction in isolated γδ T cells, it might still do so in a broader cellular context. To address this question, we analyzed IFN-γ expression in Vδ2 T cells within PBMC depleted of CD14^+^ monocytes and activated by HMBPP in the absence or presence of TLR8 ligands. The results shown in Fig. 5d indicate that the exogenous supply of IL-18 (and to a lesser extent of IL-12) indeed co-stimulated IFN-γ in HMBPP-activated γδ T cells, whereas the TLR8 ligands did not exert such co-stimulatory activity, due to the absence of monocytes in the CD14-depleted PBMC cultures. To corroborate the results obtained by intracellular flow cytometry, we measured the release of secreted cytokines IFN-γ and GrB in cell culture supernatants by ELISA. In these experiments, we compared PBMC, purified γδ T cells, and purified γδ T cells reconstituted with purified monocytes, each stimulated with TLR8 ligands only or with additional HMBPP activation. The results of three to four independent experiments are presented in Fig. 6. In PBMC, the active ligands R2152, ssRNA40 and 9.2s strongly induced IFN-γ (Fig. 6a) and GrB (Fig. 6b), both in the absence (gray columns) and in the presence (open columns) of HMBPP. The potent IFN-γ and GrB secretion in PBMC in the absence of HMBPP is most likely due to the activation of NK cells which are known producers of both cytokines, and are activated in a monocyte-dependent manner by TLR8 RNA ligands^34,38^. In line with the intracellular FACS analysis, isolated γδ T cells did neither secrete IFN-γ nor GrB. Only in the presence of monocytes in combination with the TCR stimulus HMBPP was a significant enhancing effect of active TLR8 RNAs on IFN-γ production observed, while Resiquimod showed little effect (Fig. 6a). Similarly, GrB secretion in γδ T cells was restored when monocytes were added back. Again, co-stimulation by R2152, ssRNA40 and 9.2s was observed in the additional presence of HMBPP. Baseline secretion of GrB (in the absence of HMBPP) was not enhanced by TLR8 ligands (Fig. 6b).

**Figure 7:**
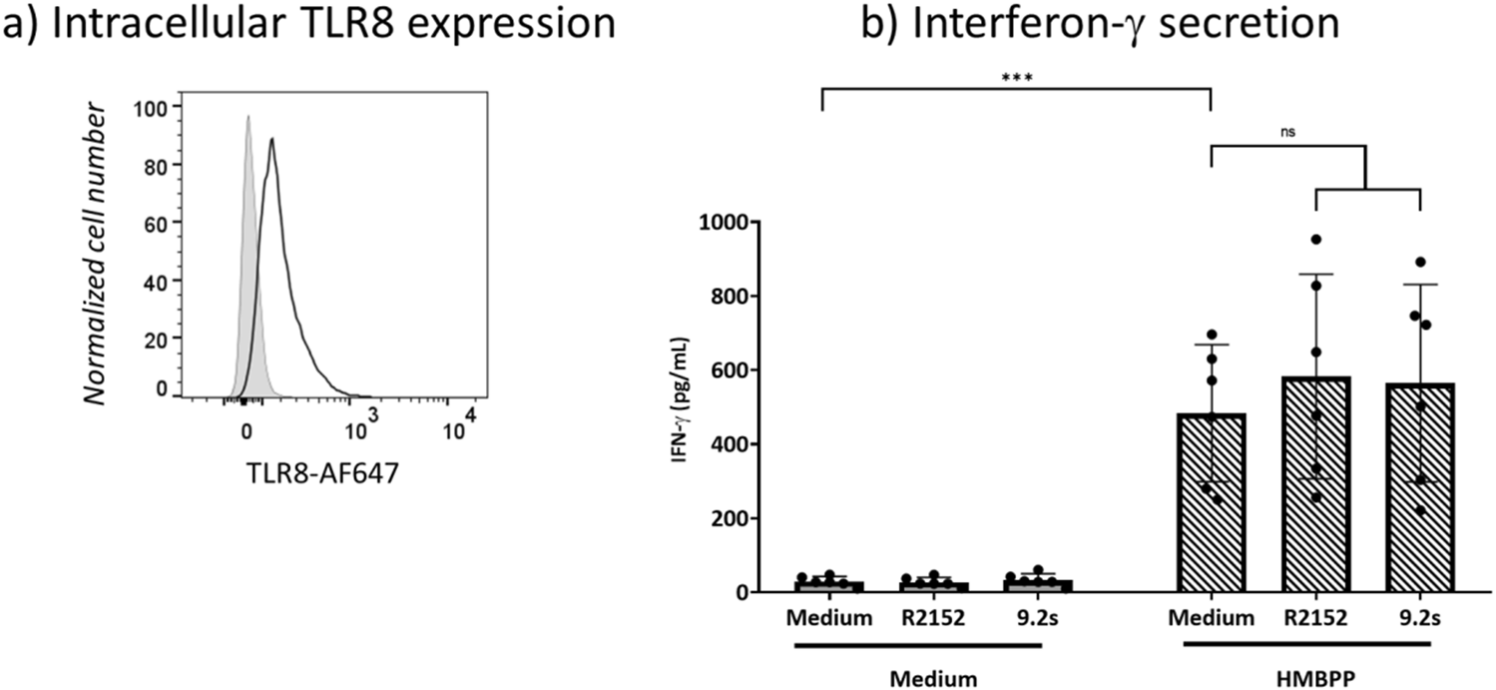
Lack of direct co-stimulation of activated γδ T cells by TLR8 RNA ligands. Short-term expanded γδ T-cell lines were generated by stimulation of PBMC with zoledronate and IL-2 and repeated addition of IL-2. After 14 days, γδ T-cell lines comprised > 94% CD3^+^Vδ2^+^ T cells. (**a**) Intracellular TLR8 expression (open histogram), isotype control (shaded histogram). (**b**) Expanded γδ T cells were cultured for 24 h in the absence or presence of 1 nM HMBPP and TLR8 RNAs R2152 or 9.2s as indicated. IFN-γ in cell culture supernatants was quantified by ELISA. All ELISAs were performed on two independent replicates in each experiment. Mean ± SD of six independent experiments are shown. Statistical significance is indicated with *** p < 0.001. ns, not significant.

The presented results indicate that freshly isolated γδ T cells are not directly co-stimulated by active TLR8 RNA ligands but require the additional presence of monocytes. Next, we asked whether pre-activated (in contrast to resting) γδ T cells might perhaps directly respond to TLR8 ligand co-stimulation. To this end, we analyzed short-term Vδ2 γδ T-cell lines which had been generated by initial stimulation with zoledronate and repeated exposure to IL-2. In comparison to circulating freshly isolated γδ T cells, expanded γδ T-cell lines expressed higher levels of TLR8 (Fig. 7a). γδ T-cell lines were then cultured for 24 h in the absence or presence of R2152 or 9.2s and without or with additional HMBPP. As shown in Fig. 7b, the RNA ligands did not induce IFN-γ secretion on their own. In the presence of HMBPP, the γδ T-cell lines secreted IFN-γ, but again the levels were not significantly modulated by the TLR8 RNA ligands.

## Discussion

The endosomal TLR7 and TLR8 which are mainly expressed in innate immune cells, are well characterized sensors of single-stranded viral and bacterial RNA. While the functional expression of TLR8 in monocytes is undisputed^28,32,38^, the functional expression of TLR7 is less clear^28^. In fact, functional TLR7 expression was more attributed to plasmacytoid DC (pDC) rather than monocytes^28^. However, more recent studies also indicate that TLR7 and TLR8 activate distinct pathways in monocytes during RNA virus infection, supporting a concept of ligand-specific regulation^36^. In addition to synthetic small molecules like Motolimod (VTX-2337), TL8-506, or Resiquimod, TLR8 specifically senses viral, bacterial, parasite (e.g., *Plasmodium falciparum*) and related synthetic RNA sequences^34,39,44^. Recently, it was discovered that TLR8 actually recognizes degradation products resulting from processing of natural or synthetic RNA molecules by endolysosomal endoribonucleases RNase T2 and RNase 2^29,45^. In view of their strong adjuvant-like activation of the innate immune arm, TLR7/8 ligands are in clinical studies mainly for oncological indications^46^. As such, TLR and other pattern recognition receptor ligands might be also valuable tools to enhance the efficacy of γδ T-cell cancer immunotherapy. Multiple approaches of γδ T-cell application in cancer immunotherapy are in development including adoptive transfer of in vitro expanded γδ T cells as well as selective in vivo activation of γδ T cells by antibodies or bispecific γδ T-cell engagers^12–14,47^. Most strategies will require additional adjuvants to counteract the immunosuppressive tumor micromilieu and/or to enhance the entry of immune cells including γδ T cells into cold tumors^48^. Along this line, it is important to explore the co-stimulation of γδ T cells by defined TLR ligands.

In this study, we used the rapid cytokine induction as a read-out to define the co-stimulatory activity of TLR7 and TLR8 RNA ligands. Previous studies have shown that ligands for TLR3, TLR4 and TLR9 co-stimulate IFN-γ induction in Vδ2 T cells within the PBMC in a monocyte- or immature DC-dependent manner^49–51^. Short-term expanded Vδ2 T cells were also found to directly respond to TLR2 ligand co-stimulation^52^, and we have previously reported direct co-stimulation of purified γδ T cells by TLR2 ligands Pam2CSK4 and FSL-1 and upregulation of TLR2 following TCR activation^53^. We also reported co-stimulation of γδ T cells by TLR3 ligand poly(I:C), in agreement with the detectable TLR3 expression in freshly isolated human γδ T cells^20,21^. We now observed a potent co-stimulatory activity of TLR8 RNA ssRNA40 and R2152^29^ as well as TLR7/8-specific RNA 9.2s^27,28^ on IFN-γ expression in Vδ2 T cells within PBMC stimulated with the pAg HMBPP. Interestingly, both the time course kinetics and the intensity of co-stimulation differed between the well characterized non-RNA TLR7/8 ligand Resiquimod and RNA ligands. The increased intracellular IFN-γ expression of HMBPP-activated γδ T cells in the presence of Resiquimod was already evident after 6 h and less obvious after 24 h, whereas the strong co-stimulatory activity of RNA ligands occurred after 24 h (Fig. 2a). The more potent activity of RNA ligands in comparison to Resiquimod also resulted in much higher levels of secreted IFN-γ and GrB measured in cell culture supernatants (Fig. 6). While the molecular explanation for the stronger co-stimulatory activity of TLR8 RNA ligands in comparison to Resiquimod requires further investigation, our results suggest that RNA ligands might be more suitable to boost γδ T-cell activation also in vivo. In contrast, TLR7-specific RNA as well as other established TLR7 ligands like Imiquimod did not have such co-stimulatory activity. However, TLR7 ligands might still modulate γδ T cell effector functions through alternative pathways, for instance by increasing the susceptibility of tumor cells to γδ T-cell mediated lysis^54^. In line with published data^34,38^, we also noticed activation of CD3-negative cells (most likely NK cells) in PBMC within the lymphocyte gate by TLR8 RNA ligands (Fig. 2b). In view of the known accessory cell dependency of TLR co-stimulation in other systems and the potent cytokine induction in monocytes by TLR8 ligands^28,32,34,36,38,49,51^, we also assumed indirect (i.e., accessory cell dependent) activation of Vδ2 T cells by TLR8 RNA ligands. This is supported by the very weak TLR8 (and TLR7) expression as determined by flow cytometry in γδ T cells when compared to monocytes. Furthermore, TLR8 RNA ligands did not co-stimulate cytokine expression in purified γδ T cells, neither in the absence (not shown) nor in the presence of TCR stimulation with HMBPP. While monocytes can produce in a stimulus-dependent manner a large variety of cytokines including TNF-α, CCL3/MIP-1α and type I interferon, we focused our attention on the important monocyte cytokines IL-1β, IL-12 and IL-18. Using neutralizing antibodies, we showed that each of these cytokines contributes to the co-stimulation of cytokine-producing Vδ2 T cells within the total PBMC population, although with slightly different potency. Considering IFN-γ, there was no statistical difference between the inhibitory effect of the anti-cytokine antibodies, even though anti-IL-12p70 was slightly less active (Fig. 4b). In the case of GrB, the inhibitory effect of anti-IL-12p70 was less significant compared to anti-IL-1β and anti-IL-18 when RNA ligands R2152 or 9.2s were used for activation (Fig. 4d). Interestingly, however, none of the three cytokines could restore the co-stimulatory effect of TLR8 RNA ligands when purified γδ T cells were activated with HMBPP, neither for IFN-γ (Fig. 5a) nor for TNF-α (not shown). Only in the case when purified γδ T cells were reconstituted with purified monocytes did we observe again the co-stimulatory activity of TLR8 RNA ligands (Fig. 5c). These results clearly demonstrate that cytokine induction in isolated Vδ2 γδ T cells cannot be elicited in the absence of monocytes (or perhaps other accessory cells like immature DC); neither HMBPP alone, nor the additional presence of TLR8 RNA ligands and/or IL-1β, IL-12 or IL-18 was able to significantly induce IFN-γ or TNF-α expression in purified Vδ2 T cells. Further experiments were designed to obtain more insight into the role of exogenous cytokines in the TLR8 RNA ligand co-stimulation. To this end, we analyzed IFN-γ induction in Vδ2 T cells within CD14 (i.e. monocyte-)-depleted PBMC. In contrast to purified γδ T cells reconstituted with monocytes, the CD14-depleted PBMC contain γδ T cells and cells other than monocytes (e.g., CD4^+^ T cells) which can also “present” HMBPP and thereby activate γδ T cells in the presence of cytokines like IL-18^55^. In this setting, we found that indeed exogenous IL-18 (and to a lesser extent IL-12) can co-stimulate IFN-γ expression in HMBPP-activated Vδ2 T cells, in the absence of TLR8 ligand co-stimulation (due to the lack of monocytes) (Fig. 5d). These results confirm that IL-18 and to a lesser extent IL-12 are important regulators of γδ T-cell activation and proliferation^56,57^ but underscore the requirement of additional activation signals.

Constitutive expression of perforin and serine proteases like GrB in circulating γδ T cells is well documented^58^. GrB and additional cytotoxic effector proteins including granulysin are important mediators of the effector function of Vδ2 γδ T cells to lyse *Plasmodium falciparum* parasites and tumor cells^59–61^. Importantly, *Plasmodium falciparum* parasites produce pAg which activate Vδ2 T cells^62^. Since RNA from *Plasmodium falciparum*-infected erythrocytes can activate TLR8^34^, the infection with *Plasmodium falciparum* parasites reflects a pathological condition where the results of our current in vitro studies might be directly relevant in vivo. As expected, we noticed strong expression of GrB in Vδ2 T cells both when analyzed within the PBMC or in isolated γδ T cells. Nevertheless, the active TLR8 RNA ligands further increased GrB expression in Vδ2 T cells within HMBPP-activated PBMC but again not in purified γδ T cells (Figs. 5,6). Interestingly, the additional co-stimulatory effect by TLR8 RNA ligands, but not the constitutive GrB expression, was abolished by neutralizing anti-IL-1β antibody (Fig. 4c,d). Like IFN-γ, GrB was secreted into cell culture supernatants when PBMC were activated only by TLR8 RNA ligands. This was also observed with purified γδ T cells reconstituted with monocytes, however at much lower levels. Taking together our results from intracellular flow cytometry and detection of secreted cytokines in culture supernatants, we conclude that TLR8 RNA ligands induce IFN-γ and GrB production within PBMC in NK cells (in a monocyte-dependent manner^34,38^) with additional contribution of γδ T cells as suggested by the increased release in the presence of HMBPP. Furthermore, purified γδ T cells require TCR activation (in our experiments by HMBPP) and the additional presence of monocytes to become sensitive to the potent co-stimulatory activity of TLR8 RNA ligands.

In contrast to the lack of direct effects on γδ T cells reported here, TLR8 ligands might exert direct co-stimulatory activity on other T-cell populations such as CD4 T cells or regulatory T cells (Treg). While the reported TLR8 expression in CD4 T cells is inconsistent^63–65^, a recent study provided clear evidence for a direct co-stimulatory activity of various TLR8 ligands on purified human CD4 T cells^65^. Moreover, since TLR8 ligation has been shown to revert the suppressive activity of Treg^64^, we have to consider that TLR8 ligands (and presumably ligands for other pattern recognition receptors as well) may modulate T-cell subsets differentially and in a microenvironment context-specific manner. Taken together, there is no doubt that TLR8 RNA ligands are highly potent co-activators of various immune cells, but the requirement of accessory cells (e.g., monocytes), additional activation signals (e.g., TCR stimulation) and specific cytokines (e.g., IL-1β, IL-18, IL-12p70) may vary between the analyzed immune cell populations (e.g., γδ T cells *versus* NK cells).

TLR ligands are in the focus of interest as adjuvants in vaccine development and cancer therapy^46,66,67^. Currently, the website https://www.clinicaltrials.gov/ lists only clinical studies with non-RNA TLR7 and/or TLR8 ligands. However, there is strong evidence that RNA ligands are potent activators of innate immunity and in consequence co-stimulators of adaptive immunity as well. At the translational level, it may require optimization of pharmacological formulation for optimal delivery of such ligands e.g. to the tumor microenvironment. The results of our present study clearly indicate that TLR8 RNA ligands provide potent co-stimulation to human γδ T cells. In view of the raising attention to bring γδ T cells into clinical application^14^, we expect that our findings will help to improve the efficacy of γδ T-cell based cancer immunotherapy.

## Supplementary Information

Supplementary Information.

## Abbreviations

BTN: Butyrophilin
GrB: Granzyme B
HMBPP: (*E*)-4-Hydroxy-3-methyl-but-2-enyl pyrophosphate
IFN-γ: Interferon-γ
pAg: Phosphoantigen
PBMC: Peripheral blood mononuclear cells
TCR: T-cell receptor
TLR: Toll-like receptor(s)
TNF-α: Tumor necrosis factor-α
