# Supplementary material for: Monocyte-dependent co-stimulation of cytokine induction in human γδ T cells by TLR8 RNA ligands
Source: Sci Rep. 2021 Jul 27;11:15231. doi: 10.1038/s41598-021-94428-6 (PMC8316369; doi:10.1038/s41598-021-94428-6)
Supplement: Supplementary file 1 — Supplementary Information. [file 41598_2021_94428_MOESM1_ESM.pdf]

Supplemental information

Monocyte-dependent co-stimulation of cytokine induction in human γδ T cells by TLR8 RNA ligands

Ruben Serrano, Christoph Coch, Christian Peters, Gunther Hartmann, Daniela Wesch, Dieter Kabelitz

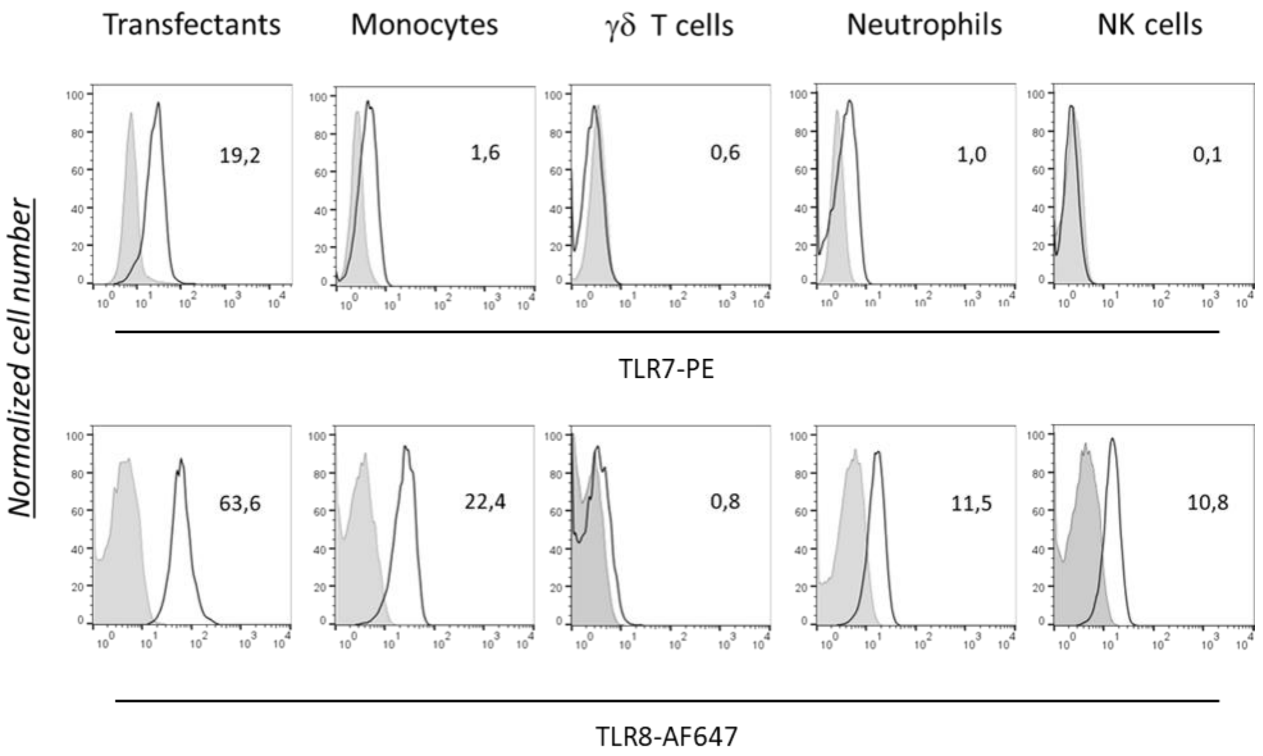

Supplemental Figure 1:

Expression of TLR7 and TLR8 in immune cells. Purified monocytes, γδ T cells, neutrophils and NK cells, as well as TLR7- and TLR8 transfectants were stained for intracellular analysis of TLR7 (TLR7-PE) and TLR8 (TLR8-AF647) expression. Histograms of a representative experiment are displayed for TLR7 in the upper panel and for TLR8 in the lower panel (open histograms). Gray histograms show the isotype controls. The numbers in the histograms indicate Δ MFI where the median fluorescence intensity (MFI) of isotype controls has been subtracted from MFI of specific staining.

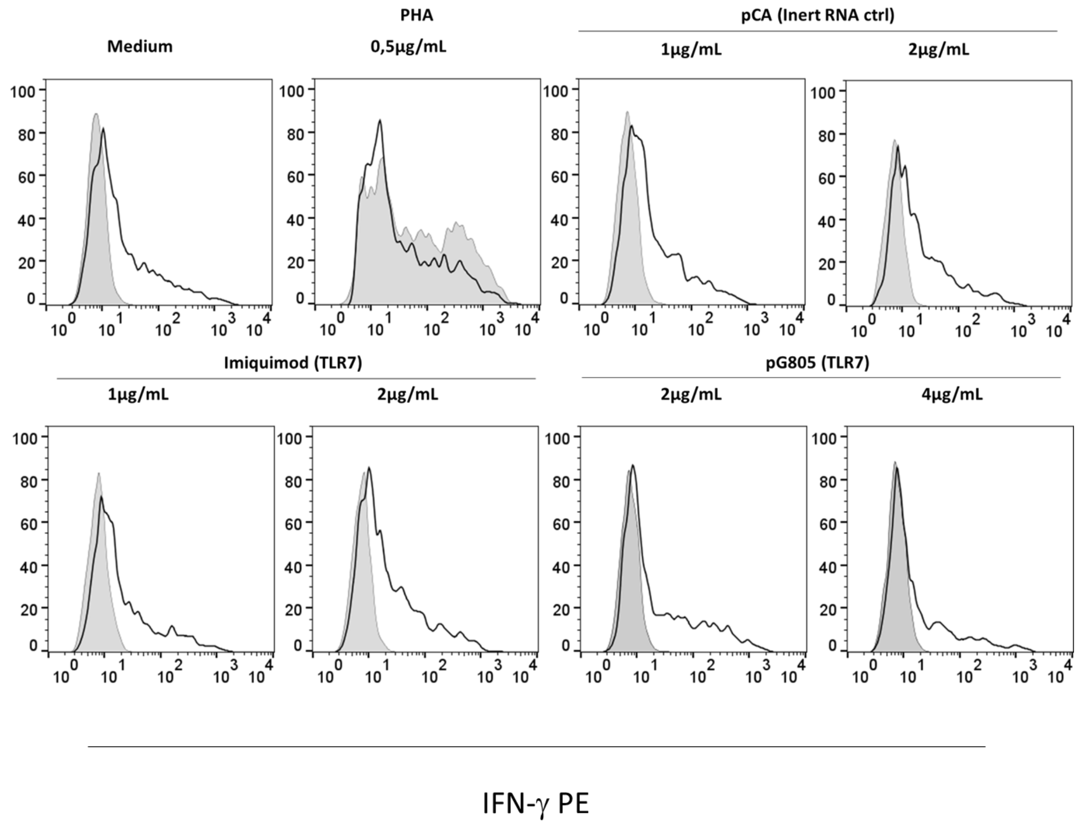

Supplemental Figure 2:

Dose titration of TLR ligands. 400.000 PBMC per well were cultured in 96-well U-bottom plates, either in the absence (gray histograms) or presence (open histograms) of the Vγ9Vδ2 T-cell selective phosphoantigen HMBPP. TLR ligands were added at indicated final concentrations. Mitogen PHA was used as a polyclonal T-cell stimulus. pCA is a negative inert RNA control. TLR7: Imiquimod, pG805. After 24 hours, cells were stained for intracellular detection of IFN-γ together with surface staining for CD3 and Vδ2. For analysis, a gate was set on $CD3^{+}V\delta2^{+}$ cells.

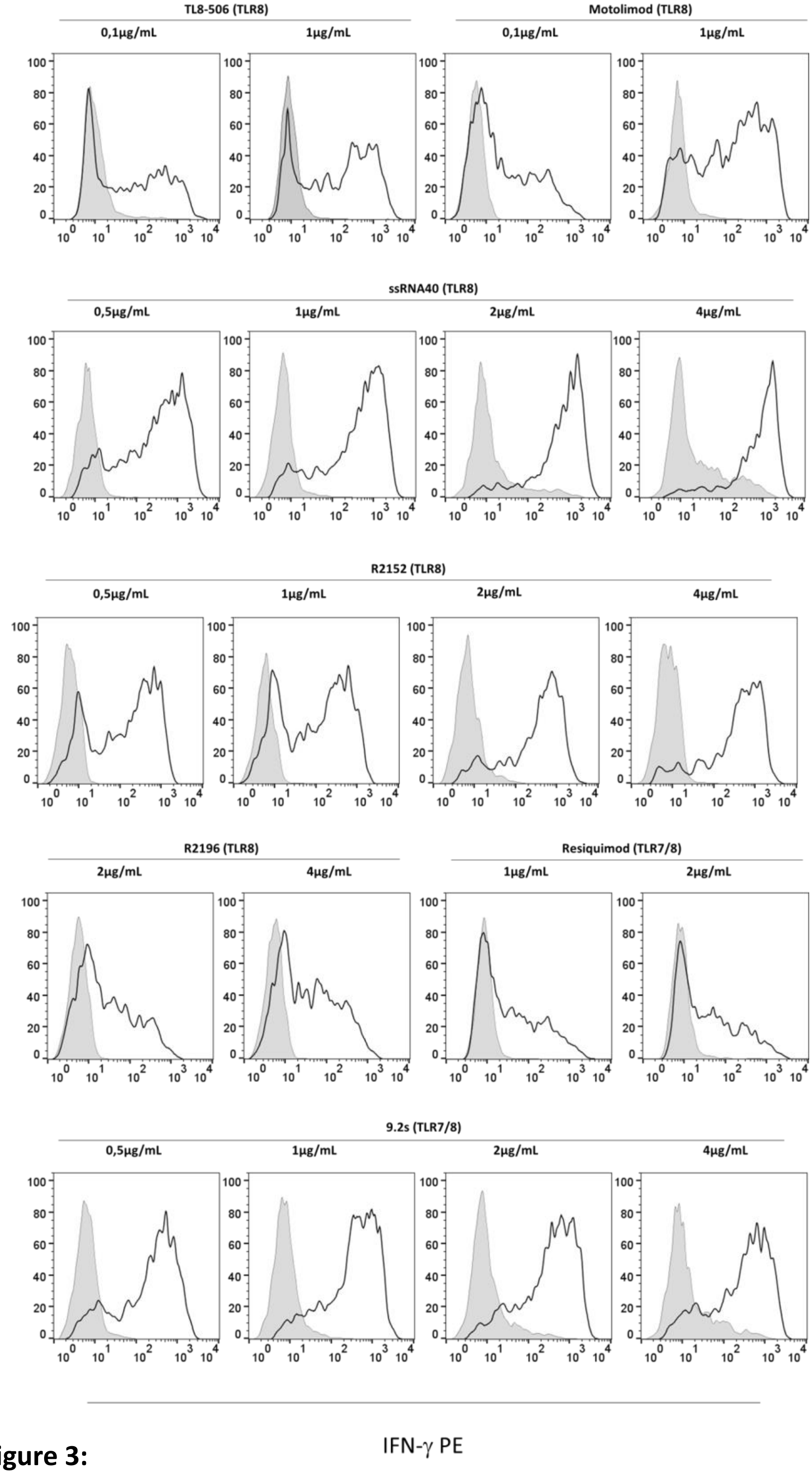

Supplemental Figure 3:

Dose titration of TLR ligands. 400.000 PBMC per well were cultured in 96-well U-bottom plates, either in the absence (gray histograms) or presence (open histograms) of the Vγ9Vδ2 T-cell selective phosphoantigen HMBPP. TLR ligands were added at indicated final concentrations. TLR8: TL8-506, Motolimod, ssRNA40, R2152, R2196; TLR7/8: 9.2s, Resiquimod. After 24 hours, cells were stained for intracellular detection of IFN-γ together with surface staining for CD3 and Vδ2. For analysis, a gate was set on $CD3^{+}V\delta2^{+}$ cells.

Supplemental Table 1

Cytokine levels in supernatants of TLR-activated monocytes

LIGANDS	CONC. FINAL	TNF-α	IL-1β	IL-12p70	IL-18
**Medium**	--	146,2	642,5	210,9	47,1
**Imiquimod**	2 µg/ml	141,9	1382,4	198,5	50,5
**ssRNA40**	2 µg/ml	8351,4	22914,2	372,2	130,1
**ssRNA41**	2 µg/ml	133,4	2318,9	235,7	50,5
**Motolimod**	0,5µg/ml	10141,2	26683,1	421,9	169,4
**Resiquimod**	2µg/ml	9966,5	26537,3	409,5	169,4

[pg/mL]
> 20.000
> 5.000
+ up to 5.000
< 1000
< 300
< 100

Purified monocytes were stimulated with the indicated concentrations of TLR7 ligand Imiquimod, TLR8 ligands ssRNA40 and Motolimod and the control ssRNA41, or TLR7/8 agonist Resiquimod. Supernatants were collected after 24 hrs and subjected to cytokine analysis using the Lumines Multiplex assay (R&D Systems, Biotechne, Wiesbaden, Germany) according to the instructions of the company. Samples were measured on a Lumines life-Match fluoroanalyzer. Concentrations are given as pg/mL. Results of one out of two experiments are shown.
